# A randomized, observer-blind, controlled phase III clinical trial assessing safety and immunological non-inferiority of Vi-diphtheria toxoid versus Vi-tetanus toxoid typhoid conjugate vaccine in healthy volunteers in eastern Nepal

**DOI:** 10.1080/21645515.2023.2203634

**Published:** 2023-04-26

**Authors:** Shipra Chaudhary, Gauri Shankar Shah, Nisha Keshary Bhatta, Prakash Poudel, Basant Rai, Surendra Uranw, Prashant Mani Tripathi, Basudha Khanal, Anup Ghimire, Nikita Rai, Birendra Prasad Gupta, Sridhar Vemula, T. Anh Wartel, Sushant Sahastrabuddhe, Tarun Saluja

**Affiliations:** a B P Koirala Institute of Health Sciences (BPKIHS), Dharan, Nepal; b International Vaccine Institute (IVI), Seoul, Korea

**Keywords:** Typhoid conjugate vaccine, Vi-DT, safety, immunogenicity, immune non-inferiority, eastern Nepal

## Abstract

Typhoid remains one of the major serious health concerns for children in developing countries. With extremely drug-resistant cases emerging, preventative measures like sanitation and vaccination, including typhoid conjugate vaccines (TCV) remain the mainstay in its prevention and control. Different types of TCVs are being developed to meet the global demand. This report outlines the results from a study done to assess the immunogenicity and safety of Vi-Diphtheria toxoid (Vi-DT) TCV in Nepal. The study was a randomized, active-controlled, immunological non-inferiority and safety study. Eligible participants from Sunsari and Morang districts of eastern Nepal were randomized into 4 study groups (A-D) within 3 age strata (6 months to <2 years, 2 to <18 years, and 18 to 45 years). Groups A to C received a single dose (25 μg) of Vi-DT test vaccine from any of the 3 lots, while group D received the comparator, Typbar-TCV®, Vi-tetanus toxoid (Vi-TT) vaccine (25 μg) in 1:1:1:1 ratio and evaluated at 4 weeks postvaccination with 6 months follow-up. Amongst 400 randomized participants, anti-Vi-IgG seroconversion rates for all age strata in Vi-DT pooled groups (A+B+C) were 100.00% (97.5% CI 98.34–100.00) vs 98.99% (97.5% CI 93.99–99.85) in Vi-TT group (D) at 4 weeks. Comparable safety events were reported between the groups. Three serious adverse events (1 in Vi-DT; 2 in Vi-TT group) were reported during the 6 months follow-up, none being related to the investigational product. Thus, Vi-DT vaccine is safe, immunogenic, and immunologically non-inferior to Vi-TT when analyzed at 4 weeks postvaccination.

## Introduction

Typhoid, a bacterial infection caused by *Salmonella typhi*, remains one of the common enteric diseases in developing countries.^1–3^ The World Health Organization (WHO) estimates 11–20 million cases of typhoid fever per year, resulting in about 128,000–161,000 deaths annually.^4^ It is an important cause of morbidity and mortality, especially in children living in poor communities that lack safe water and adequate sanitation.^3,5^ Although preventable and treatable, the emergence and rising trends of extremely antimicrobial resistant typhoid cases makes treatment difficult, lengthy, and expensive.^1,6^ Preventive measures like water sanitation and hygiene (WASH) and vaccination are thus critical steps in controlling this disease.^7^ WHO recommends typhoid vaccination for high-risk groups and for outbreak control.^3^ Many countries, including Nepal, have recently incorporated the typhoid conjugate vaccine in their national routine immunization programs.^8^

At present, the vaccines available for typhoid are of 3 main types- Vi polysaccharide (ViPS) vaccine, typhoid conjugate vaccine (TCV), and live attenuated oral Ty21a vaccine. Conjugation, i.e. coupling to protein carriers, transforms the T cell-independent polysaccharide vaccines to T cell-dependent antigenic vaccines and thus enhances their immunogenicity and utility in infants and younger children.^9^ Carrier proteins like genetically modified cross-reacting material (CRM) of diphtheria toxin, tetanus toxoid (TT), diphtheria toxoid (DT), Hemophilus influenza protein D (HiD), and meningococcal outer membrane protein complex (OMPC) have been used for inducing immunologic memory, herd immunity, and reduction of nasopharyngeal colonization for better prevention of various diseases.^9,10^ Similarly, preclinical studies have shown polysaccharide intracellular adhesion (PIA)-rSesC (Staphylococcus epidermidis surface exposed) protein conjugate vaccine candidate could protect against S. epidermidis infection.^11^ Thus, conjugate vaccine would be the preferred option among the various typhoid vaccines.

In January 2018, WHO prequalified the first conjugate vaccine for typhoid, Typbar TCV® in age 6 months and above.^1,2,12,13^ Currently, four TCVs from different sources using different carrier proteins for conjugation are commercially available in India – PedaTyph™ (Bio-Med Ltd, India), ZYVAC TCV™ (Zydus Cadila, India), Typbar TCV® (Bharat Biotech India Ltd, Hyderabad), and TYPHIBEV® (Biological E Ltd, India). (2) Amongst these, two vaccines- Typbar TCV ® and TYPHIBEV® are WHO prequalified and available in Nepal through private market and Gavi procurement, respectively.

The protective efficacy of a single dose of Vi polysaccharide-tetanus toxoid (Vi-TT) conjugate vaccine (Typbar TCV) as part of Typhoid Vaccine Acceleration Consortium (TyVac) in Nepal was above 80% in first year and above 70% in second year.^12^ TYPHIBEV®, consisting of Vi polysaccharide from Citrobacter freundii WR7011 conjugated to CRM_197_, a nontoxic mutant of diphtheria toxoid, has also demonstrated comparable safety and immunogenicity to Typbar TCV.^14^

The International Vaccine Institute (IVI) developed and transferred technology to SK Bioscience (Seoul, South Korea) for a TCV consisting of diphtheria toxoid conjugated purified Vi polysaccharide from S Typhi strain C6524 (Vi-DT), targeting WHO prequalification and aid in the global supply of TCVs. The results of phase 1 and 2 studies of the Vi-DT vaccine conducted in the Philippines have demonstrated safety good immunogenicity.^15,16^ The overall results of the main phase 3 study done at 4 sites in Nepal showed Vi-DT vaccine to be safe and immunologically non-inferior as compared to Vi-TT.^2^ Herein, we report age stratum specific immunogenicity and safety details of a single dose of Vi-DT versus a single dose of Vi-TT vaccine at 4 weeks after vaccination among the subset of study participants enrolled at B.P. Koirala Institute of Health Sciences (BPKIHS) Dharan, a tertiary care center in eastern Nepal.

## Materials & methods

This was a randomized, observer-blind, active-controlled, non-inferiority, phase 3 clinical trial to compare the immunogenicity and safety of Vi-DT vaccine (SK Bioscience) to the locally available licensed Vi-TT vaccine Typbar TCV (Bharat Biotech International). It was done over a period of one year (2019–2020) at BPKIHS, Dharan, Nepal. Regulatory approvals were obtained from Nepal Health Research Council (Nepal), Department of Drug Administration (Nepal), Institutional Review Board of IVI (Korea) and the Institutional Review Committee at BPKIHS. This study is part of the phase 3 clinical trial conducted at four different sites in Nepal.^2^

Eligible participants were healthy individuals aged 6 months to 45 years volunteers. Individuals with an acute or chronic illness that could interfere with interpretation of the study endpoints, those with known history of typhoid or household contact with or intimate exposure to an individual with typhoid or had previously received a typhoid vaccine or who were already involved in any other clinical trial were excluded. Written informed consent or assent was obtained from all participants, parents, or legal guardians before participation in the screening activities with a copy of the document given to them for their records.

Eligible participants from different villages of Sunsari and Morang districts of eastern Nepal were randomized into four study groups (groups A – D) in a 1:1:1:1 ratio and within the groups separated into 3 age strata (6 months to <2 years, 2 years to <18 years, and 18 years to 45 years). Groups A-C received a single dose of Vi-DT test vaccine composed of 25 µg Vi polysaccharide/0.5 ml, presented in 3 ml multiple dose glass vial from one of the 3 lots (lot numbers S121901, S121902, and S121903). Group D received a single dose of the comparator Typbar-TCV® vaccine composed of 25 µg Vi polysaccharide/0.5 ml, presented in a single dose glass vial (lot number 76C19023A).

Randomization was done using block randomization process, with random block sizes of four and eight for ensuring effective balance between the interventions. An independent statistician generated the randomization list with unique sequential numbers for each participant and vaccination group (A – D). The blinded study team was provided with separate randomization list without information on vaccine allocation for enrollment while the blinded study team had the randomization list with vaccine allocation information for vaccine administration.

All the randomized participants underwent baseline blood sampling followed by administration of 0·5 mL of either Vi-DT or Vi-TT vaccine by the unblinded study nurse in a separate room. Participants received intramuscular injection in the left anterolateral thigh (for participants below 2 years of age) or the left deltoid region and were observed at the study site for a minimum of 30 minutes after vaccination. The participants, investigators, and clinical staff involved in postvaccination safety evaluation were all blinded to group assignment.

Post-vaccination adverse events were recorded in a diary card in the form of immediate reactions (within 30 minutes), solicited adverse events (during the first 7 days), unsolicited adverse events (till 4 weeks), and serious adverse events (throughout the 24-week study period). For safety evaluation, weight, height, heart rate, respiratory rate, and body temperature of each participant were taken and recorded at each study visit. All adverse events were classified by system organ class and preferred terms of the Medical Dictionary for Regulatory Activities (version 22.0). The assessment of vaccine safety and monitoring of safety events was done by an independent data safety monitoring board, which included independent clinical experts and a biostatistician.

For immunogenicity analysis, blood samples were taken from each participant before vaccination at baseline (day 0) and after vaccination at day 28 (week 4) and day 168 (week 24). An in-house ELISA was used to measure anti-Vi IgG antibody concentrations.^2,16,17^ The antibody titers were expressed in international units [IUs] per mL relative to the WHO international standard reference panel from the National Institute for Biological Standards and Control (NIBSC 16/138; Appendix B, p. 8), with the lower limit of 0·14 IU/mL.

The primary endpoint of this study was anti-Vi IgG seroconversion rates of Vi-DT vaccine (pooled groups A – C) compared to Vi-TT vaccine (group D) at 4 weeks postvaccination. Seroconversion was defined as a four-fold or greater increase in antibody titers in comparison to the baseline titers. Anti-Vi IgG seroconversion rate at 24 weeks post-vaccination was taken as the secondary endpoint.

Safety endpoints were frequency of local and systemic adverse events (immediate, unsolicited, solicited, or serious) reported in the study.

Sample size calculation was done based upon 99% power for determining non-inferiority of immunogenicity of Vi-DT vaccine (pooled groups A-C) compared to Vi-TT vaccine (group D); the details of the sample size calculation for the main study have been mentioned elsewhere. (2) Based upon the WHO Technical Report Series 924, the predefined non-inferiority margin of −10% was taken and non-inferiority of the Vi-DT vaccine compared to Vi-TT vaccine was considered if the lower limit of 97.5% CI for the difference between the seroconversion rates was above this limit. A one-sided test of non-inferiority was used with a significance level of 0.0125.^2,18^

Data were analyzed using both intention-to-treat and per-protocol analysis methods; the definitions for the analysis sets are mentioned elsewhere.^2^ Immunogenicity analysis set was used for the primary immunogenicity endpoints and the full analysis set for demographics and safety endpoints, descriptively summarized by group and age strata.

For statistical analysis, SAS 9.4 statistical software was used along with chi-square tests for determining p-value for categorical variables.

This trial is registered with ClinicalTrials.gov, NCT03933098.

## Results

**Figure 1.**
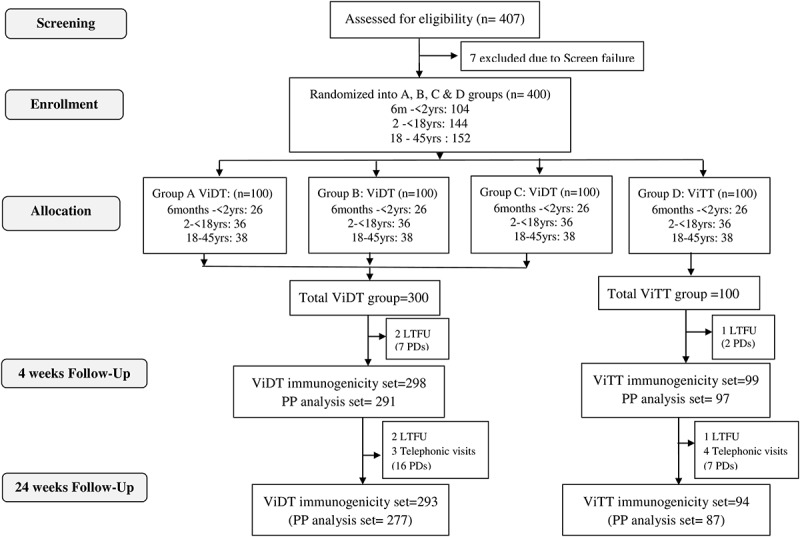
CONSORT flow diagram showing trial profile.

From 08 Dec 2019 to 01 Mar 2020, a total of 407 healthy volunteers aged 6 months to 45 years were screened, amongst which 7 were excluded due to screening failure (preexisting medical conditions). Four hundred participants were enrolled and randomly assigned to groups A, B, C, or D (100 participants in each group), with a total of 300 subjects in Vi-DT test group and 100 subjects in Vi-TT comparator group as shown in the CONSORT flow diagram in Figure 1.

The full analysis set included all 400 subjects, while the immunogenicity analysis set at 4 weeks follow-up included 397 subjects (298 in Vi-DT and 99 in Vi-TT groups respectively) as 3 participants were lost to follow-ups (LTFU). In the per-protocol analysis set, there were a total of 388 subjects (291 and 97 in Vi-DT and Vi-TT groups, respectively). There were 9 protocol deviations (PDs) observed due to visit deviations mostly because of travel restrictions associated with COVID-19 pandemic. Immunogenicity data were collected from 387 subjects in the final 24-week follow-up as detailed in Figure 1.

The demographic distribution of the study participants was balanced across all the groups as shown in Table 1.

**Table 1.** Demographic distribution of the study participants (n = 400).

	Vi-DT Group	Lot 1	Lot 2	Lot 3	Vi-TT Group	Total	
Characteristics	N = 300	N = 100	N = 100	N = 100	N = 100	N = 400	*p*-value
Male (%)	155 (51.67)	53(53.00)	47(47.00)	55(55.00)	52 (52.00)	207(51.75)	.9539*
Female (%)	145 (48.33)	47(47.00)	53(53.00)	45(45.00)	48(48.00)	193(48.25)	
Mean (SD)	15.80 (12.56)	15.71 (12.14)	15.72 (12.59)	15.97 (13.05)	15.12(12.28)	15.63 (12.48)	.6378^#^
Median (min, max)	15.05 (0.51, 44.69)	14.62 (0.51, 43.85)	15.61 (0.53, 44.59)	15.02 (0.59, 44.69)	13.14 (0.61, 41.01)	14.58 (0.51, 44.69)	

*Chi square test; ^#^Two sample t test; Vi-DT = Vi-Diphtheria toxoid; Vi-TT = Vi-Tetanus toxoid.

The anti-Vi IgG seroconversion rate at 4 weeks after single dose vaccination in all age strata was 100.00% (97.5% CI 98.34 to 100.00; all 298 participants) in Vi-DT group as compared to 98.99% (97.5% CI 93.39 to 99.85; 98 of 99 participants) in Vi-TT group, with the difference between the proportions being 1.01 (97.5% CI −3.07 to 5.09) with the lower limit of the 97·5% CI for the difference being greater than non-inferiority margin of −10% (Table 2).

**Table 2.** Anti-Vi IgG seroconversion rates in Vi-DT vaccine vs Vi-TT vaccine at 4 weeks postvaccination: Immunogenicity set.

4 Weeks Postvaccination	Vi-DT Group		Vi-TT Group		Vi-DT - Vi-TT	*p*-value^‡^
	n/N	Seroconversion rate (97.5% CI)	n/N	Seroconversion rate (97.5% CI)	Difference (97.5% CI)^†^	
All Ages	298/298	100.00 (98.34, 100.00)	98/99	98.99 (93.39, 99.85)	1.01 (−3.07, 5.09)	.7857
Age Strata1 (6months- <2years)	78/78	100.00 (95.31, 100.00)	26/26	100.00 (87.13, 100.00)	0.00 (0.00, 0.00)	NA
Age Strata2 (2 - <18 years)	107/107	100.00 (96.53, 100.00)	36/36	100.00 (90.36, 100.00)	0.00 (0.00, 0.00)	NA
Age Strata3 (18 - <45 years)	113/113	100.00 (96.71, 100.00)	36/37	97.30 (86.18, 99.52)	2.70 (−3.51, 8.92)	.2467

^†^Generalized linear model for binomial distribution; ^‡^Generalized linear model for binomial distribution and Fisher’s exact test for age strata 3 p-value; Vi-DT= Vi-Diphtheria toxoid; Vi-TT = Vi-Tetanus toxoid; CI = Confidence Interval.

In the per-protocol analysis set, the anti-Vi IgG seroconversion rates of Vi-DT vaccine (pooled groups A-C) were non-inferior toVi-TT vaccine (group D) in all age strata (1.03 [97.5% CI −3.10 to 5.17]) and in each individual age stratum (Appendix A).

At 24 weeks postvaccination, anti-Vi IgG seroconversion rate was 98.98% (95% CI 97.03 to 99.65; 290 of 293 participants) in Vi-DT group as compared to 97.87% (95% CI 92.57 to 99.41; 92 of 94 participants) in Vi-TT group in all age strata. Individual age stratawise seroconversion rates in Vi-DT group were 98.65% (92.73–99.76; 73 of 74) in 6 months to less than 2 years, 100.00% (96.53–100.00; 107 of 107) in 2 to less than 18 years, and 98·21% (93.72–99.51; 110 of 112) in 18 to 45 years participants. The seroconversion rates in Vi-TT group were 100.00% (84.54–100.00; 21 of 21) in 6 months to less than 2 years, 100.00% (90.36–100.00; 36 of 36) in 2 to less than 18 years, and 94·59% (82.30–98.50; 35 of 37) in 18 to 45 years participants. Similar seroconversion rates were observed in the per protocol analysis set (Appendix B).

Among the 300 participants in Vi-DT vaccine group, 11 (3.67%) reported immediate reactions during first 30 mins postvaccination; of them, 10 (3.33%) were local reactions and 1 (0.33%) systemic reaction. Among the 100 participants in Vi-TT vaccine group, 3 (3.00%) reported immediate reactions during first 30 mins postvaccination, all 3 (3.33%) being local reactions. Common local reactions in both the vaccine groups were pain, tenderness, and redness at the injection site, while the most common immediate systemic reaction was headache. The number of participants reporting solicited adverse reactions within 7 days post-vaccination were 101 (33.67%) participants in Vi-DT group with 83 (27.67%) reporting local and 41 (13.67%) reporting systemic events and 44 (44.00%) participants in Vi-TT group with 37 (37.00%) reporting local and 14 (14.00%) reporting systemic adverse reactions. Most of the systemic adverse events reported in both the vaccine groups were fever, headache, vomiting, and diarrhea. These were mild to moderate in severity and resolved within a few days. At 4 weeks follow-up, there were 63/300 (21.00%) and 18/100 (18.00%) participants reporting unsolicited events in Vi-DT and Vi-TT group, respectively. The most common unsolicited adverse events reported in the study were diarrhea, vomiting, fever, cough, and nasopharyngitis. They were mild to moderate in severity with none being related to the vaccine and resolved without any sequelae. During the 24 weeks study period, there were a total of 3 serious adverse events: 1 (0.33%) in Vi-DT vaccine group and 2 (2.00%) in Vi-TT group. These included 1 (0.33%) medical termination of pregnancy in Vi-DT group, 1 (1.00%) medical termination of pregnancy in Vi-TT group, and 1 (1.00%) tubercular pleural effusion in Vi-TT group. Both of the participants undergoing medical termination of pregnancy had a negative urine pregnancy test prior to enrollment and were counseled regarding contraception during screening but had spontaneous pregnancy and opted for termination within 4 weeks follow-up. Right sided tubercular pleural effusion was reported in a 19-year-old male after 63 days postvaccination with Vi-TT, for which he required hospitalization and 9 days of inpatient care with continued anti-tubercular drug treatment post hospital discharge. None of these SAEs were related to the study vaccines as judged by the site investigators and the data safety monitoring board. The age stratawise details of the adverse events are presented in Table 3.

**Table 3.** Adverse events profile of participants after vaccination with Vi-DT vs Vi-TT vaccine.

	Vi-DT Group (*N* = 300)			Vi-TT Group (*N* = 100)			*p* value
	Overall	Local AE, n/N** (%)	Systemic AE n/N** (%)	Overall	Local AE n/N** (%)	Systemic AE n/N** (%)	
**Immediate reactions within 30 minutes postvaccination**							
All ages	11/300 (3.67%)	10 (3.33%)	1 (0.33%)	3/100 (3.00%)	3 (3.00%)	0 (0.00%)	1.00^#^
Age strata 1 (6months- <2years)	4/78 (5.13%)	4 (5.13%)	0 (0.00%)	1/26 (3.85%)	1 (3.85%)	0 (0.00%)	1.00^#^
Age strata 2 (2 - <18 years)	2/108 (1.85%)	1 (0.93%)	1 (0.93%)	2/36 (5.56%)	2 (5.56%)	0 (0.00%)	.26^#^
Age strata 3 (18 - <45 years)	5/114 (4.39%)	5 (4.39%)	0 (0.00%)	0/38 (0.00%)	0 (0.00%)	0 (0.00%)	.33^#^
**Solicited adverse events within 7 days after vaccination (related to vaccine)**							
All ages	101/300 (33.67%)	83 (27.67%)	41 (13.67%)	44/100 (44.00%)	37 (37.00%)	14 (14.00%)	.07*
Age strata 1 (6months- <2years)	27/78 (34.62%)	18 (23.08%)	14 (17.95%)	12/26 (46.15%)	7 (26.92%)	7 (26.92%)	.29*
Age strata 2 (2 - <18 years)	38/108 (35.19%)	34 (31.48%)	10 (9.26%)	19/36 (52.78%)	17 (47.22%)	4 (11.11%)	.06*
Age strata 3 (18 - <45 years)	36/114 (31.58%)	31 (27.19%)	17 (14.91%)	13/38 (34.21%)	13 (34.21%)	3 (7.89%)	.84*
**Unsolicited adverse events within 4 weeks after vaccination**							
All ages	63/300 (21.00%)			18/100 (18.00%)			.51*
Age strata 1 (6months- <2years)	35/78 (44.87%)			13/26 (50.00%)			.64*
Age strata 2 (2 - <18 years)	14/108 (12.96%)			3/36 (8.33%)			.56*
Age strata 3 (18 - <45 years)	14/114 (12.28%)			2/38 (5.26%)			.36*
**Serious adverse events during entire study period**							
All ages	1/300 (0.33%)			2/100 (2.00%)			.15^#^
Age strata 1 (6months- <2years)	0 (0.00%)			0 (0.00%)			NA
Age strata 2 (2 - <18 years)	0 (0.00%)			0 (0.00%)			NA
Age strata 3 (18 - <45 years)	1/114 (0.88%)			2/38 (5.26%)			.15^#^
Respiratory, thoracic and mediastinal disorders Tubercular pleural effusion	0 (0.00%) 0 (0.00%)			1 (1.00%) 1 (1.00%)			.25^#^
Surgical and medical procedures Medical termination of pregnancy	1 (0.33%) 1 (0.33%)			1 (1.00%) 1 (1.00%)			.43^#^

*Chi-square test; ^#^Fisher’s exact test; n = number of participants reporting the adverse event; N = total number of participants; AE = Adverse Events; Vi-DT = Vi-Diphtheria toxoid; Vi-TT = Vi-Tetanus toxoid.

## Discussion

This study shows that a single dose of Vi-DT vaccine is safe and immunologically non-inferior to the comparator Vi-TT vaccine at 4 weeks postvaccination in the studied age groups.

We found no significant difference in anti-Vi IgG seroconversion rates between Vi-DT vaccine (groups A-C) and Vi-TT vaccine (group D) at 4 weeks (100.00% vs 98.99%) and 24 weeks (98.98% vs 97.87%). Immune non-inferiority of Vi-DT vaccine compared with Vi-TT vaccine at 4 weeks postvaccination was proven with the lower limit of 97.5% CI of difference between the seroconversion proportions being −3.07%, which was greater than the predefined non-inferiority margin of −10%. The proportions of participants reporting adverse events after vaccination were comparable in Vi-DT and Vi-TT groups with no significant difference.

There are various types of typhoid conjugate vaccines being studied in different population and are at various stages of development.^13,19^ Several studies have reported safety and immunogenicity of Vi-TT Typbar TCV^R^.^12,20–23^ Similarly, efficacy, safety, and immune response to longevity of another Vi-TT vaccine Pedatyph^TM^ (Bio-Med Pvt Ltd, India) has been shown in different studies in India.^24,25^ Comparative studies have reported similar immunogenicity and safety of ZyVAC TCV (Cadila Healthcare Ltd, India) as compared to Typbar TCV.^26^ TYPHIBEV®, the latest WHO-prequalified TCV was also shown to be immunologically non-inferior to TypbarTCV with comparable safety and reactogenicity in healthy infants, children, and adults.^14^ A phase II preliminary report from a study on a novel Vi-diphtheria toxoid TCV (manufactured by Bio Farma, Indonesia) demonstrated that Vi-DT vaccine was safe and immunogenic in children 2–11 years old.^27^ Clinical phase I and II studies of Vi-DT TCV (manufactured by SK Bioscience, Korea) in Filipino adults, followed by children and infants, have reported its safety and immunogenicity.^15,16^ The results from the main study of the phase III trial in Nepal demonstrated Vi-DT TCV immunogenicity in Nepalese population.^2^ The phase III trial involving participants aged 6 months to 45 years in the Philippines demonstrated immune-equivalence and safety of multi-dose and single-dose formulations of the Vi-DT vaccine, providing further evidence for the consideration of its WHO prequalification.^28^

The results from the present study demonstrating non-inferior immunogenicity and safety of single dose Vi-DT vaccine versus the licensed WHO prequalified Vi-TT vaccine with comparable results in various age strata add further evidence to support its licensure and WHO prequalification, and thereby, add to the current supply chain and diversity of TCV portfolio to meet global demands.

Our study was affected by the ongoing COVID-19 pandemic. One of the limitations was the follow-up visits being affected by the respective travel restrictions, causing many predefined visits to not be conducted within the visit window, which in turn increased the number of protocol deviations. However, these protocol deviations had minor effects on the per-protocol analyses. Lot to lot consistency of the different lots of Vi-DT vaccine could not be commented upon from this sub-study and has been demonstrated in the main study. (2) In this study, we could not study the immune noninterference of Vi-DT typhoid conjugate vaccine with measles-rubella vaccine due to unavailability of sufficient number of eligible participants during the study period. Additionally, as Nepal is a typhoid-endemic country, there was a possibility of enrolling individuals with subclinical infection or individuals who were asymptomatic carriers of typhoid affecting this study. However, there would have been equal distribution of any potential sources of error among the 4 randomized groups, thus not affecting the statistical comparisons.

In conclusion, our study shows that a single dose of Vi-DT test vaccine has seroconversion rates similar to Vi-TT Typbar TCV^R^, thus proving its immunogenicity, safety, and immunological non-inferiority in 6 months to 45 years old individuals. This study helps to support the licensure and WHO prequalification of the studied Vi-DT typhoid conjugate vaccine.

## Appendices Appendix A: Anti-Vi IgG seroconversion rates of Vi-DT vaccine vs Vi-TT vaccine at 4 weeks postvaccination: Per Protocol set

**Table:**

Week 4 Postvaccination	Vi-DT Group (Pooled Groups A+B+C)		Vi-TT Group		Vi-DT – Vi-TT	*p*-value^‡^
	n/N	Seroconversion rate (97.5% CI)	n/N	Seroconversion rate (97.5% CI)	Difference (97.5% CI)^†^	
All Ages	291/291	100.00 (98.30, 100.00)	96/97	98.97 (93.27, 99.85)	1.03 (−3.10, 5.17)	.7851
Age Strata1	72/72	100.00 (94.93, 100.00)	24/24	100.00 (86.20, 100.00)	0.00 (0.00, 0.00)	NA
Age Strata2	107/107	100.00 (96.53, 100.00)	36/36	100.00 (90.36, 100.00)	0.00 (0.00, 0.00)	NA
Age Strata3	112/112	100.00 (96.68, 100.00)	36/37	97.30 (86.18, 99.52)	2.70 (−3.54, 8.94)	.2483

^†^Generalized linear model for binomial distribution with group and strata as covariates; ^‡^Generalized linear model for binomial distribution with group and strata as covariates for p-value for all ages; Fisher’s exact test for p-value for each age strata; Vi-DT = Vi-Diphtheria toxoid; Vi-TT = Vi-Tetanus toxoid; CI = Confidence Interval.

## Appendix B: Anti-Vi IgG seroconversion rates of Vi-DT vaccine vs Vi-TT vaccine at 24 weeks postvaccination: Per Protocol set

**Table:**

Week 24 Post Vaccination	Vi-DT Group (Pooled Groups A+B+C)		Vi-TT Group		*p*-value^‡^
	n/N	Seroconversion rate (95% CI)	n/N	Seroconversion rate (95% CI)	
All Ages	274/277	98.92 (96.86, 99.63)	85/87	97.70 (92.00, 99.37)	.8301
Age Strata1	69/70	98.57 (92.34, 99.75)	20/20	100.00 (83.89, 100.00)	1.0000
Age Strata2	102/102	100.00 (96.37, 100.00)	34/34	100.00 (89.85, 100.00)	NA
Age Strata3	103/105	98.10 (93.32, 99.48)	31/33	93.94 (80.39, 98.32)	.2418

^‡^Generalized linear model for binomial distribution with group and strata as covariates for p-value for all ages; Fisher’s exact test for p-value for each age strata; Vi-DT = Vi-Diphtheria toxoid; Vi-TT = Vi-Tetanus toxoid; CI = Confidence Interval.
